# Biomedical Data Manifest: A lightweight data documentation mapping to increase transparency for AI/ML

**DOI:** 10.1038/s41597-026-06670-0

**Published:** 2026-02-11

**Authors:** Daniel Bottomly, Christopher G. Suciu, Benjamin Cordier, Nathaniel Evans, Alfonso Poire, Christina Zheng, The ARTNet Consortium, Jeffrey Myers, Vlad Sandulache, Trever Bivona, Jack Roth, Boyi Gan, Albert Koong, Pankaj Singh, Michael Hollingsworth, Jixin Dong, Brian Druker, David W. Goodrich, Song Liu, Tao Liu, Christopher Willey, Joshi Alumkal, Keith Syson Chan, Phuoc Tran, Chunru Lin, Erina Vlashi, Alice Soragni, Paul C. Boutros, Erik Knudsen, Agnieszka Witkiewicz, Xingxing Zang, Michael Deininger, Jeffrey W. Tyner, Alan Hutson, Shannon K. McWeeney, Jeffrey W. Tyner, Alan Hutson, Shannon K. McWeeney

**Affiliations:** 1 Knight Cancer Institute, Oregon Health & Science University, Portland, OR USA; 2 Department of Pathology and Laboratory Medicine, Oregon Health & Science University, Portland, OR USA; 3 Division of Hematology & Medical Oncology, Knight Cancer Institute, Oregon Health & Science University, Portland, OR USA; 4 Department of Cell, Developmental and Cancer Biology, Knight Cancer Institute, Oregon Health & Science University, Portland, OR USA; 5 Roswell Park Comprehensive Cancer Center, Buffalo, NY USA; 6 Division of Oncological Sciences Oregon Health & Science University, Portland, OR 97239 USA; 7 Department of Head and Neck Surgery, University of Texas, MD Anderson Cancer Center, Houston, TX USA; 8 Bobby R. Alford Department of Otolaryngology, Baylor College of Medicine, Houston, TX USA; 9 Department of Medicine, University of California at San Francisco, San Francisco, CA USA; 10 Department of Thoracic and Cardiovascular Surgery, The University of Texas MD Anderson Cancer Center, Houston, TX USA; 11 Department of Experimental Radiation Oncology, Division of Radiation Oncology, The University of Texas MD Anderson Cancer Center, Houston, TX USA; 12 Department of Radiation Oncology, Division of Radiation Oncology, The University of Texas MD Anderson Cancer Center, Houston, TX USA; 13 Department of Oncology Science, University of Oklahoma Health Sciences Center, Oklahoma City, OK USA; 14 OU Health Stephenson Cancer Center, University of Oklahoma Health Sciences Center, Oklahoma City, OK USA; 15 Eppley Institute for Research in Cancer and Allied Diseases, University of Nebraska Medical Center, Omaha, NE USA; 16 Department of Urology, Roswell Park Comprehensive Cancer Center, Buffalo, NY USA; 17 Department of Biostatistics and Bioinformatics, Roswell Park Comprehensive Cancer Center, Buffalo, NY USA; 18 Department of Radiation Oncology, University of Alabama at Birmingham, Birmingham, AL USA; 19 Division of Hematology and Oncology, University of Michigan Rogel Cancer Center, Ann Arbor, MI USA; 20 Department of Urology, Neal Cancer Center, Houston Methodist Research Institute, Houston, TX USA; 21 Department of Radiation Oncology, University of Maryland School of Medicine, Baltimore, MD USA; 22 Department of Molecular and Cellular Oncology, The University of Texas MD Anderson Cancer Center, Houston, TX USA; 23 University of California, Los Angeles, CA USA; 24 Department of Orthopaedic Surgery, University of California, Los Angeles, Los Angeles, CA USA; 25 Department of Molecular and Cellular Biology, Roswell Park Comprehensive Cancer Center, Elm and Carlton Street, Buffalo, NY USA; 26 Department of Microbiology and Immunology, Albert Einstein College of Medicine, Bronx, NY USA; 27 Versiti Blood Research Institute, Milwaukee, WI USA

**Keywords:** Research data, Medical research, Data publication and archiving

## Abstract

Biomedical machine learning (ML) models raise critical concerns about embedded assumptions influencing clinical decision-making, necessitating robust documentation frameworks for datasets that are shared via external repositories. Fairness-aware algorithm effectiveness hinges on users’ prior awareness of specific issues in the data – information such as data collection methodology, provenance and quality. Current ML-focused documentation approaches impose impractical burdens on data generators and conflate data/model accountability. This is problematic for resource datasets not explicitly created for ML applications. This study addresses these gaps through a two-step process: First, we derived consensus documentation fields by mapping elements across four key templates. Second, we surveyed biomedical stakeholders across four roles (clinicians, bench scientists, data manager and computationalists) to assess field importance and relevance. This revealed important role-dependent prioritization differences, motivating the development of the Biomedical Data Manifest – a modular template employing persona-specific field presentation reducing generator burden while ensuring end-users receive role-relevant information. The Biomedical Data Manifest improves transparency for datasets deposited in public or controlled-access repositories and bias mitigation across ML applications.

## Introduction

The adoption of artificial intelligence and machine learning (AI/ML) approaches for predictive tasks in biomedicine is rapidly expanding, influencing decisions that can directly affect clinical care and resource allocation. High-quality training data are therefore essential for developing reliable and equitable models. However, most large-scale biomedical datasets were not originally designed for machine learning applications, making them vulnerable to hidden confounders and biases. Well-documented harms have already emerged from such issues, including stereotypes in word embeddings^1^ structural biases in large language models^2^, proxy variables in healthcare management^3^, and demographic disparities in facial recognition^4^. Although a variety of methods have been proposed to detect and mitigate bias, these strategies are most effective when potential issues such group imbalance are known in advance^5^. Robust metadata standards and transparent dataset documentation are thus critical to enable systematic bias assessment and to promote fairness and reproducibility across biomedical AI studies^6^.

As the size and complexity of publicly available biomedical datasets increases, details regarding the formation and composition of a given dataset remain sparse or spread amongst the methods or supplementary documents in a number of publications or websites. These gaps are particularly problematic for datasets that are deposited in public or controlled-access repositories, where repository-level metadata alone is often insufficient to convey critical contextual information needed for responsible reuse. Supplemental documents, often in PDF form, also are generally not computable and can be lost over time. It is also often difficult to match details discussed in a given manuscript to available data and metadata that is shared or deposited to public repositories.

Dataset documentation templates (also known in the literature as transparency templates^7^) have emerged as a potential solution in the era of machine learning and artificial intelligence (AI) to improve transparency across the data life cycle and ensure responsible AI practices. These templates, such as Datasheets for Datasets^6^, Data Cards^8^, and HealthSheets^7^ arose from the need to provide structured summaries of essential details about the lineage and provenance of machine learning datasets, including their motivation, composition, collection process, and intended uses (common data documentation categories). Each associated category has either structured or unstructured elements depending on the template. In practice, these documentation artifacts are intended to accompany datasets that are shared via external repositories or portals, providing a richer context than repository metadata fields alone.

**Fig. 1:**
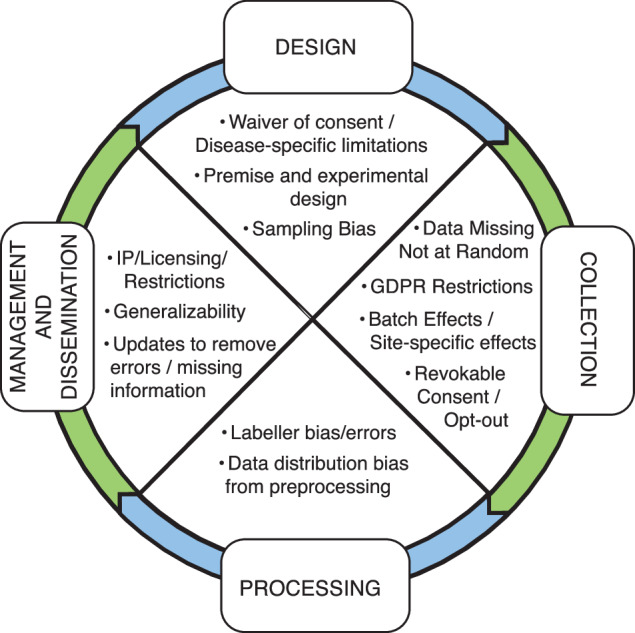
Dataset documentation can improve transparency across the data lifecycle. The four main stages of the data lifecycle are shown in the boxes connected in order with notched arrows in a circular path. In the middle of the circle are the corresponding fields that can be gathered as part of data documentation to help provide context for future use.

Data documentation can make explicit the appropriate uses of data, issues in data collection and considerations for dissemination and re-use throughout the data life cycle (Fig. 1). However, despite the clear importance of data documentation, uptake has been inconsistent. A key reason for this is domain and organizational culture challenges. In particular, templates are often not suitable for some data contexts; data documentation approaches are often ad hoc; and not integrated into existing data generation workflows^9^. From Heger *et al*.’s^9^ evaluation of ML practitioners, seven design requirements were identified: explicit connection between data documentation and responsible AI implications, need for practicality and utility given time demands, adaptability to different data contexts, feasibility of simple automation for some metadata, clear target audience, standardization and centralization, and integration into existing tools and workflows. Our premise is that to increase uptake further, mapping across templates is critical to define core consensus elements for a given domain (in this case: biomedical data). Further, we believe the relevance of these elements is contextual based on personas (data generator -bench or clinician scientist-, computationalist, data steward etc.) that have different roles in data and model life cycles. The knowledge of these lifecycles is likely heterogenous and shaped by experience/perspective (e.g., How data was generated vs impact on downstream modeling). Another challenge is the post-hoc creation of data documentation artifacts. In this work, we focus on the subset of documentation activities that support preparation of datasets for external sharing and reuse through public or controlled-access repositories, rather than on internal data management practices within laboratories or institutions. As formal data documentation is a relatively new concept, especially in the biomedical sciences, almost no publicly available datasets currently have them, outside of those datasets specifically designed for AI/ML (e.g. Bridge2AI).

To address these issues, we first carried out a field-level mapping across commonly used data documentation templates. We then performed a survey encompassing participants working in common biomedical research roles. From the results of this survey, we created a new data documentation format termed the ‘Biomedical Data Manifest’ in addition to an accompanying template and code to simplify creation. The name was chosen to draw a connection with the manifest in logistics which lists all the items in a shipment and is essential for the transportation process. Manifests are used to ensure transparency, accuracy, and compliance throughout the shipping process. Our proposed Biomedical Data Manifest was designed to simplify the process and burden of documentation for data generators to meet similar goals (transparency, accuracy, and compliance) for the dissemination process.

## Methods

### Mapping across data documentation templates

We reviewed four data documentation templates: DataCards^8^, DescribeML^10^, Data Sheets for Datasets^6^ and HealthSheets^7^. These were selected because (i) they are widely cited in the ML and AI literature, (ii) they provide detailed, publicly available question sets suitable for field-level mapping, and (iii) they are not direct derivatives of one another (for example, opendatasheets^11^ is based off of Datasheets for Datasets and was therefore excluded as redundant). Other templates that are either specific to particular data types^12^ or focused primarily on output structure^13^ rather than field content were not included. We first highlighted common data documentation categories and their associated elements and the stages of the data life cycle that they address (Supplementary Table S1). As we utilized the discrete fields across each template, DataSheets for Datasets, was reviewed but not formally part of the mapping of each element across the templates. One challenge we found when comparing fields between the data documentation approaches was the apparent differences in terminology. To address this, we first created a glossary that included the definitions of a given term for each of the three approaches (if available) as well as the definition we would use going forward (Supplemental Table S2). One important example of this was the term ‘Instance’ which was used in all three approaches and explicitly defined both for DescribeML and HealthSheets in the accompanying manuscripts. DescribeML defined an ‘Instance’ in terms of a group of entity attributes which differed from its definition in HealthSheets and the implied definition in the DataCards as an observation. We decided to use the latter definition due to its consistency with usage in biomedical research. Based on our glossary, we created a mapping between the captured fields/concepts from each of the three approaches (Supplemental Table S3). From this, we formed a set of 136 consensus fields incorporating the novel contributions of each data documentation approach while removing redundancies. From this list we divided the consensus fields into seven main categories: General Information, Uses of Data, Composition, Ethical Legal and Social Issues (ELSI), Provenance and Lineage, Labeling Provenance and Lineage as well as Maintenance and Distribution. We further reduced this list to 100 fields, after collapsing related fields, removing clear redundancies and enforcing separation of concerns between data and model (e.g. removing fields that should be part of Model Cards^14^ or similar; Supplemental Table S4).

### Consensus field prioritization survey

We conducted a survey over 6 months involving investigators across the Acquired Resistance to Therapy network (ARTNet) consortium of 5 academic medical centers^15^ which is focused on research related to the mechanistic study of cancer recurrence as well as acquired resistance to cancer therapies. The OHSU Institutional Review Board (IRB) determined (OHSU IRB #27457) that this project does not constitute research involving human subjects as defined under 45 CFR 46.102(e). The activity involved the collection and analysis of survey data that were recorded in such a manner that no identifiable private information was obtained and the identities of respondents could not be readily ascertained by the investigators, directly or indirectly through identifiers or codes. Because there was no intervention or interaction with living individuals for research purposes and no use of identifiable private information, the project does not meet the regulatory definition of human subjects research and therefore is not subject to the requirements of 45 CFR 46 or ongoing IRB oversight. No written informed consent under human subjects regulations was required for analysis of the anonymous survey responses. We asked study participants to review our consensus fields and rank them based on perceived importance given their roles and experience interacting with biomedical data. In order to decrease the time commitment for the participants, we did not ask them to rank the 17 ‘General Information’ fields as well as added 4 ‘pivot’ questions which allowed the skipping of sections if they weren’t relevant based on the participants research and/or experience (Supplemental Fig. S1). Testing of the survey prior to deployment indicated that depending on familiarity and role the expected time to completion ranged from 5 to 45 minutes for participants. For each field, we asked participants to indicate whether they thought the field was: Essential, Less Essential, Non-Essential or Redundant or alternatively whether it had an Unclear Meaning. These rankings were assigned weights of 5, 3, 0 or N/A respectively. Our relevance score to assess each question was then the average across participants for a given group/persona (N/A values were excluded when computing). To provide us with some additional context, we also added 3 additional informational questions about their role and how data is handled with respect to preparation and submission of datasets to external repositories or archives. Analysis of this data was performed using R (v4.4.2) including tidyverse (v2.0)^16^, and openxlsx (v4.2.7.1) with figures generated using ggplot2 (v3.5.1), patchwork (v1.3.0) and ggrepel (v0.9.6).

### Approaches for Re-use

To maximize usage, we are providing three key components of the Biomedical Data Manifest. For the first: the final fields were implemented in Microsoft Forms as an example data manifest collection tool that could be shared and adapted as needed^17^ and an editable copy of the Biomedical Data Manifest collection form is provided as Supplementary File S1, as well as in our Github repository (See Code Availability section). For the second, we provide an example of the manifest survey result file format^18^. The third, to aid in rendering the interactive manifest is an HTML template using Bootstrap (v4.6.2) and jQuery (v3.5.1) along with a new R package ‘BioDataManifest’^19^ that will facilitate the creation of Biomedical Data Manifest files from a given manifest collection result file, as well as from hosted data on the Genomic Data Commons^20^ and dbGaP^21^–supporting manifest generation specifically for datasets that are already curated and shared outside the originating institution.

## Results

**Fig. 2:**
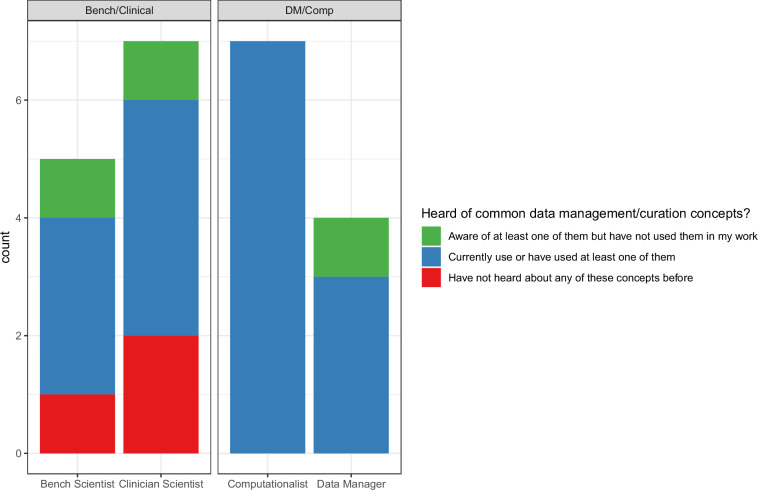
Summary of survey participant roles. A survey of 23 participants was conducted with each participant assigning themselves a role. We further grouped these roles into two ‘aggregate personas’. Shown is the count (Y-axis) of roles (X-axis) relative to each persona (facet text). In addition, the count of each role was further divided by how well the participants knew some common concepts about data management/curation (color).

**Fig. 3:**
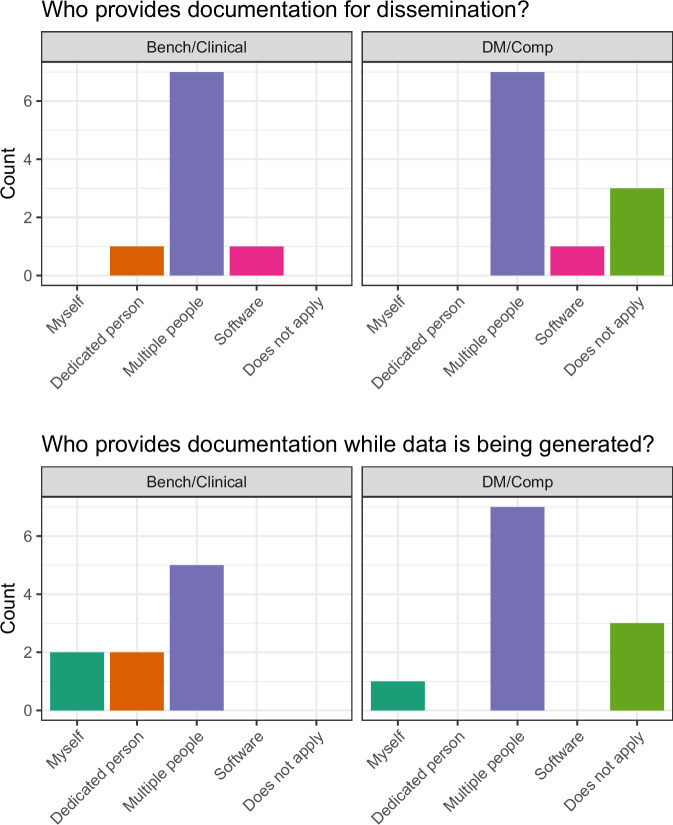
Self-reported responsibilities for data documentation in the survey. Each survey participant was asked about which lab/team member was responsible for data documentation during collection (top) as well as during dissemination (bottom). There were four multiple choice answers as well as an ‘Does not apply’ field (X-axis). The count of participants is shown on the Y-axis. Note that participants who had not previously heard of data documentation approaches are not counted at this step (resulting in n = 20).

In order to gain insight into the most important fields from our data documentation consensus mapping for biomedical research, we carried out a survey asking participants at each of the ARTNET centers to rank the 83 fields not considered to be part of the ‘General Information’ category (Supplemental Table S5). A total of 27 participants took part in the survey. However, we excluded four participants who answered fewer than 25/90 questions and who would not have been excluded from the initial ‘pivot’ question. Survey results of the remaining 23 participants are provided in Supplemental Table S5. As part of this survey, we also gathered information on the participant’s role, whether they have heard of common data management/curation concepts (Metadata, Data documentation templates, FAIR, CARE, TRUST) as well as how data was managed in their lab/team both during collection as well as during dissemination. Our participants mostly consisted of ‘Clinician Scientists’ and ‘Computationalists’ (14/23) with the remainder split between ‘Data Managers’ (4/23) and ‘Bench Scientists’ (5/23) leading us to group them into two ‘aggregate personas’: Bench/Clinical and DM/Comp with roughly equal number of participants. This also reflects for the most part, where they are in the data lifecycle with Bench/Clinical most commonly generating the data and DM/Comp most commonly managing and analyzing it. Three participants were unaware of the common data management/curation concepts and so were excluded from the survey (Fig. 2). Of the remaining 20 participants, the majority reported that data documentation duties were split between multiple people both while data generation was ongoing as well as after completion of the data set (Fig. 3).

**Fig. 4:**
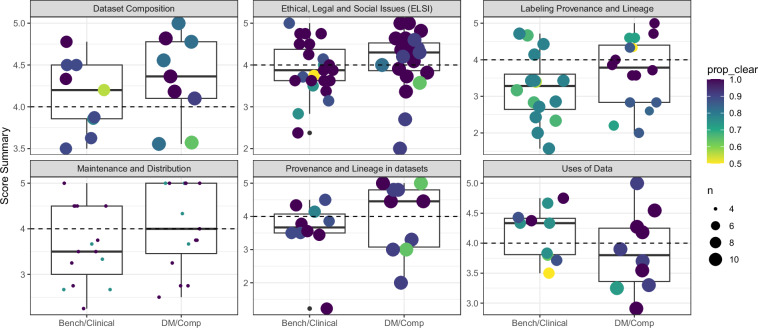
Average survey scores for each persona grouped by category. For each persona (X-axis), the average score (Y-axis) is shown for the corresponding fields/concepts (dots) across all available survey participants. Separate subplots (facets) are shown for each category. Size of the dots indicates the number of participants, while color of the dots indicates the proportion of the participants who chose a ranking as opposed to indicating that the field/concept was unclear. The dashed line indicates out cutoff of 4.0 for field/concept relevance.

As described in the **Methods**, the average ranking score was calculated for each field across all the participants providing a means to prioritize each field based on the participant’s rating of a field’s importance/essentiality. We assessed the distribution of the scores for the six categories assessed in the survey (Supplemental Fig. S2) and decided to compute per aggregate persona (providing ‘relevance’ for those personas; Fig. 4).

**Fig. 5:**
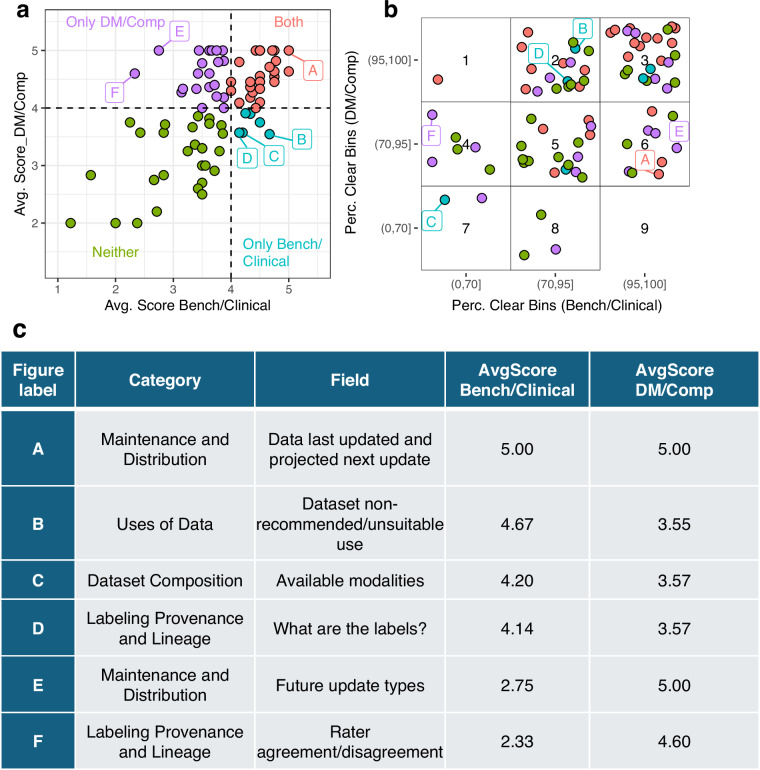
Categorization of participant scores. The relevance scores for each field was summarized for both the aggregate personas. (**a**) Using a cutoff of 4.0, the fields (points) were separated into four groups (colors and dashed lines) based on the relevance for each aggregate persona (X and Y-axis). Six fields are highlighted and labeled A-F. (**b**). For the corresponding fields, the percentage of participants who did not consider the field to be unclear are shown (dots). We classified the percentages into three groups for each aggregate persona (delimited by dashed lines) and labeled each ‘sector’ by a number to simplify referencing. Again, the percentages are relative to the aggregate personas (X and Y-axis) and are colored and labeled as in (**a**). (**c**) The fields labeled on (**a**) and (**c**) are shown along with their categories and corresponding figure label.

While importance/essentiality highlights the value of a field, the relevance score allows us to evaluate how applicable the score is for specific personas. Based on the distribution of the scores overall as well as the distribution of the relevance scores (for each of the aggregate personas) we selected an empirical cutoff of 4.0 for prioritization. Of the 83 fields, 48 were considered to be relevant for the DM/Comp aggregate persona group, while 33 were considered to be relevant for the Bench/Clinical aggregate persona group. Of these, 27 overlapped between the two (Fig. 5a). In particular, one field ‘Data last updated and projected next update’ was scored as a 5.0 for both aggregate personas likely indicating the perceived importance of communicating update frequencies for those participants who actively update data releases (Fig. 5a**; annotated as ‘A’**). In addition, there were a number of fields that differed in relevance between the aggregate personas. For instance, only six fields were considered to be relevant only to the Bench/Clinical group: ‘Dataset non-recommended/unsuitable use’, ‘Available modalities’, ‘What are the labels?’ (Fig. 5a**annotated as ‘B’-‘D’**) as well as ‘Dataset recommended/suitable use’, ‘Intentionality of sensitive human attribute collection’ and ‘What has changed on update?’. On the other hand, 21 fields were only relevant to the DM/Comp group. The two with the largest differences were ‘Future update types’ and ‘Rater agreement/disagreement’ (Fig. 5a**; annotated as ‘E’, ‘F’**). Interestingly, these fields received relevance scores below 3.0 for the Bench/Clinical group indicating a large gap in perceived relevance.

In addition to the average relevance score, the fraction of participants who indicated the question had Unclear Meaning was also deemed important as it indicated that the terms should be refined. In practice we defined the ‘Percentage Clear’ as the percentage of participants for whom a given term was understood enough to be ranked. It was not always the case that the highest scoring fields were the clearest to the participants (Fig. 5b); in particular only ¾ participants in the DM/Comp provided a ranking for the field ‘Data last updated and projected next update’ (Percentage Clear of 75%) which had been scored as a 5.0 for both aggregate personas. Overall, only 12/27 fields deemed relevant for both groups also had Percent Clear of 95% or greater (Fig. 5b**Sector 3**). We considered these twelve fields to be the ‘core’ fields that were consistently highly ranked as well as understood. Conversely, of the 29 fields neither group considered to be relevant, only 6 of them had Percent Clear of 95% or greater in both groups (Fig. 5b**Sector 3**) suggesting that in general these fields were not understood–contributing to their low scores. In addition, of the 21 uniquely relevant fields to the DM/Comp group, less than half (8 /21) had a Percentage Clear of at least 95%, similarly this was also the case (2/6 fields) for the uniquely relevant fields to the Bench/Clinical group. In total 32 fields were considered relevant to at least one aggregate persona but had a Percent Clear <95%. We proceeded to modify these fields for clarity and/or with additional examples (Supplemental Table S6).

While ranking the fields, participants could also provide questions/comments related to each field (Supplemental Tables S5, S6). We observed some common themes in the comments, in particular one participant noted that much of the information on the survey could be covered through a combination of a data-centric publication such as a Data Descriptor^22^ and Model Cards^14^. Another participant noted that there was a natural hierarchy to many of the fields and we recognized that leveraging this hierarchy could reduce the burden of completing a data manifest.

Based on our survey results, we created a lightweight HTML-based document that we termed the Biomedical Data Manifest. An example template for filling out a Biomedical Data Manifest is available as a Microsoft Forms survey^17^ as well as an editable document of form fields (see Data Availability) for data generators, who are preparing datasets for deposition in public or controlled-access repositories. These templates incorporated the 17 General Information fields along with the 54 relevant fields from the survey as well as also enforcing a hierarchy supplemented by 2 additional pivot questions. The results from this template could then be rendered into the Biomedical Data Manifest HTML file for ease of sharing and dissemination using our freely available R package ‘BioDataManifest’. In the HTML Manifest template, fields can be filtered or highlighted according to persona-specific relevance scores, allowing DM/Comp users to foreground fields related to data lineage, update frequency, and labeling processes, while Bench/Clinical users can emphasize intended use, contraindicated uses, and clinical constraints.

Finally, since it can be difficult to find information on the Biomedical Data Manifest fields for publicly available large biomedical datasets, we also provide functionality within our R package to query Genomic Data Commons^20^ and the Database of Genotypes and Phenotypes (dbGaP)^21^. This allows the creation of a partial Biomedical Data Manifest for either aggregate persona based on the available data in these external repositories, aligning the template with real-world conditions of repository-based data sharing.

## Discussion

We created a consensus mapping from three commonly used data documentation approaches resulting in 100 fields which were grouped into 7 main categories. A survey was devised to determine how essential each field was to biomedical researchers categorized by four common roles (personas): ‘Clinician Scientists’, ‘Computationalists’, ‘Data Managers’ and ‘Bench Scientists’. We aggregated the personas into two groups ‘Bench/Clinical’ and ‘DM/Comp’ roughly matching their position in the data lifecycle. One limitation is that our sample size for the survey was relatively small—23 participants. This was likely due to the burden of ranking 83 fields across (at most) five of the six categories. We attempted to reduce this burden further through the use of pivot questions which allowed participants to skip categories that were not relevant to their role or experience. This approach even allowed three participants to skip ranking fields and exit the survey since they had never heard of any of the common data management/curation concepts.

Before the survey we asked several general information questions about who in their lab/team performed data documentation during data generation as well as during data dissemination. Interestingly, the majority of the survey participants indicated that multiple people were charged with documenting datasets in either scenario. As this was seen in both DM/Comp and Bench/Clinical groups, this likely reflects a mixture of both not having dedicated staff for data management and instead relying on the individual researchers and shared resources at their institution. Another potential contributing factor is the possibility of blurred roles in computational or large resource generating labs (e.g., data curator, data standards, data manager, data disseminator). Our survey questions did not have the specificity to allow us to elucidate that further. We recognize that there are potential sources of bias due to the small sample size (n = 23), skew in distribution of responses by role and that the fact that participation was limited to investigators and staff within the ARTNet consortium. Therefore, this study should be viewed as formative research with the goal of carrying out further larger scale studies in the future covering additional consortiums/institutions, fields and roles within biomedical research to improve generalizability.

It is important to note that the roles we chose reflected our translational research-oriented consortium. The focus of this paper is on biomedical datasets not intended originally for AI/ML use while the templates we used in the mapping were not specific to translational research but could be used in other contexts such as population health, with the addition of any distinct roles that are necessary for those fields.

The ranks of each survey field were scored creating both a relevance score for each aggregate persona as well as an indication of how well understood the question was, termed the ‘Percentage Clear’. Using a fixed cutoff for the relevance score, we found that there were notable differences in the fields chosen by either of the aggregate personas. For instance, only six fields were considered to be relevant in the Bench/Clinical group but not in the DM/Comp group. One of these was the field ‘Dataset non-recommended/unsuitable use’ which could be considered a vague field with one interpretation being the data generator’s opinion on data while another could be highlighting regulatory or compliance limitations on the use of the dataset such as language in human subjects research consent forms indicating disease-specific limitations on research (e.g. only leukemia research is allowed). Further highlighting the potential ambiguity is that all DM/Comp group participants ranked the field on average as moderate to low importance while only ~75% of the Bench/Clinical group were clear enough on the meaning to rank it. Note that 32/83 fields were considered relevant by a given group but had <95% of respondents that were clear on its meaning. We modified these fields for clarity, however, future surveys could be conducted to determine the efficacy of the new version. Additionally, 21 fields were uniquely relevant to the DM/Comp group. One interesting example was ‘rater agreement/disagreement’ which could indicate that members of the Bench/Clinical view high levels of disagreement as common place due to the often-subjective nature of the interpreting biomedical data such as images etc. Another example was the ‘Future update types’ field which could reflect perceived differences in how and when a dataset is updated. For example, there are set rules for updating clinical trials^23^ whereas other large scale (possibly longitudinal) databases such as TCGA^24^ or BeatAML^25^ commonly used by those in the DM/Comp group may be updated in a more ad hoc (to the end user) manner. These differences between the aggregate personas suggest there are potential gaps in understanding how to characterize the complexity of the data and account for its potential uses.

This highlights the importance of moving towards a more standardized terminology to facilitate communication between individuals with varying biomedical disciplines and even across fields. Having a common vocabulary is only part of the foundation; teams also need a clear, skills-based and inclusive responsibility framework that explains who participates in each step of the documentation lifecycle and which capabilities they bring to that work. In the modern data documentation lifecycle, clarity around roles and responsibilities is essential for collaboration, quality, and accountability, particularly for the stages that culminate in preparing and depositing datasets into external repositories. However, traditional frameworks like the RACI chart (which indicates who is Responsible, Accountable, Consulted and Informed), while useful, can sometimes feel too rigid or hierarchical for data teams that thrive on flexibility and shared ownership. What is needed is a skills-aware responsibility framework, inspired by RACI but tailored to data work, that supports clarity without exclusion and helps everyone understand who contributes, how they contribute, and when their input matters. Regardless, the Biomedical Data Manifest provides the flexibility to be customized, and incorporating team input from multiple perspectives is critical for ensuring that relevant fields are included.

We also found that 29 of the 83 fields were not considered to be relevant to either aggregate persona. One such example was ‘Distribution of sensitive human attributes (e.g. histograms or barplots)’. Such plots are often included in data documentation templates but tend to increase the size and complexity of the final product as well as be very subjective in nature, neither of which is desirable for a standard template or computability in general. This results in distribution plots being shown in relatively few public data documentation instances. For example, they were absent in 3/4 Data Card examples as part of the Data Cards Playbook^26^. We recognize that the non-relevant fields for our survey participants may be relevant for other roles/personas or in other contexts.

An important limitation of our Biomedical Data Manifest is that such templates can never be completely prescribed, given the observed variation in experience amongst the different personas. Our results demonstrate that the perceived importance and clarity of documentation fields vary systematically across personas, reflecting their position in the data lifecycle and their typical responsibilities. This finding reinforces that the Biomedical Data Manifest should not be a static template but a dynamic framework that is persona-dependent, adapting to highlight fields and terminology most relevant to the contributing roles. Practically, this is realized in the HTML Manifest template by allowing customization facilitating persona-specific filtering and highlighting of fields, so that computational/data-management users see lineage and update-focused content, while bench and clinical users see fields emphasizing intended use, contraindications, and clinical constraints. For example, fields emphasizing data lineage and computation provenance may be prioritized in DM/Comp-authored manifests, whereas contextual and usage-specific fields (e.g., regulatory limits, intended clinical use) may be emphasized in Bench/Clinical-authored manifests. This persona-driven adaptability transforms the Biomedical Data Manifest from a static reporting form into a flexible, role-aware framework that evolves with the scientific context, fostering reproducibility, interoperability, and meaningful data reuse. The Biomedical Data Manifest author would need license to create new personas or tweaks on existing ones with the idea that the authoring personas will ultimately drive metadata that is collected and recorded with reference to how the dataset should be used. For instance, datasets generated for technology development should only be used for validation, not development of diagnostics.

In the survey results, it was noted that all of the fields in the section “Labeling Provenance and Lineage” and the field “Known application and Benchmarks” seemed to blur data documentation (from perspective of data generators) with model cards. This is not surprising given that the original templates were created by computationalists and used for datasets intended for specific AI/ML tasks. It is important to note that Model Cards were originally defined as “short documents accompanying trained machine learning models that provide benchmarked evaluation in a variety of conditions”^14^. While both labeling and benchmarking may be critical for datasets created in a machine learning context (relative to specific models or resources), these may have little to no relevance for data generators working with general datasets or resources.

**Fig. 6:**
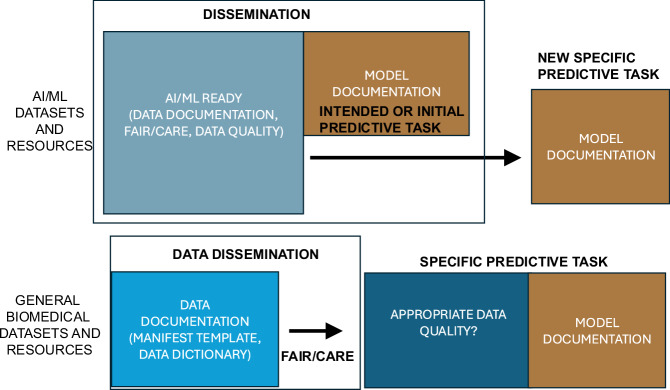
To improve transparency and uptake of data documentation templates, we propose that in the context of AI/ML datasets and resources (top row), data documentation templates are not sufficient as the goal should be to have data that is “AI/ML Ready”. For general biomedical data sets (bottom row), documentation is streamlined using the Biomedical Data Manifest as there is no intended predictive task for these data sets at time of generation.

Recent work from the NIH Common Fund Bridge2AI project on AI/ML readiness highlights that data documentation is key for pre-model explainability but not alone sufficient for assessment for intended AI/ML predictive task^27^. This distinction is important, as it allows us to (1) further delineate between the dataset and downstream modeling and (2) allows distinction between datasets created for specific AI/ML task versus general resources and datasets. Therefore, we view this as a shift from the “historical” use of one-size-fits-all data documentation templates to explicit use for either data documentation templates for AI/ML datasets and resources (Data Sheets, Health Sheets etc.) or templates for general biomedical datasets and resources that were not created specifically for AI/ML but could still be used for training models (Biomedical Data Manifest) (Fig. 6).This reduces a tremendous burden on the data generator and can lead to more relevant documentation.

At present, depending on the journal, editors do have requirements for authors to provide some detailed documentation regarding both the data and the methods utilized (e.g. the Reporting Summary for Nature and STAR methods for Cell). For journals that require deposition of datasets in external community or generalist repositories, structured dataset-level documentation can complement repository records by clarifying provenance, intended use, and limits of reuse. However, these requirements are clearly hybridizing both elements of the data and analytical approaches and are often not readily computable. This is likely due to the common paradigm of authors both providing datasets as well as reporting analyses in a given publication. Even in journals specifically focused on data sets such as Nature’s Scientific Data and Elsevier’s Data in Brief, where there has been in-depth analysis on how to enhance “AI-readiness” (see Giner-Miguelez *et al*.^28^), this conflation of data and analytical details persists. Our recommendation is to separate data documentation, designed to travel with data shared via repositories and support reuse, from model documentation such as Model Cards^14^ that references trained models. This is because, although model quality is dependent on the quality of the training data, datasets themselves can be used for many different purposes if effectively documented^29^. As noted in prior work that we move towards more computable and integrated approaches for documentation to reduce the burden on data generators. We note if these templates meet FAIR guidelines as a findable resource (with a DOI attribution etc.), they can be attributed similar to publications in progress reports and grants as markers of productivity^30^ providing a further incentive for data generators. By design, the Biomedical Data Manifest is a repository-oriented documentation framework intended to travel with datasets shared via public or controlled-access repositories and complement existing repository metadata and journal data-sharing requirements, rather than to manage internal laboratory data flows.

## Supplementary information

Supplementary File S1

Supplementary Figures

Table S1, Table S2, Table S3, Table S4, Table S5, Table S6

## Data Availability

All data supporting the findings of this study are available within the paper and its Supplementary Information. In particular, Supplementary Table S2 contains the glossary, Supplementary Tables S3, S4 contain details on the data documentation consensus mapping and Supplementary Tables S5, S6 contains the survey results. The Biomedical Data Manifest collection form is also provided as Supplementary File S1 and in the Github repository (see below).

## Code availability

The ‘BioDataManifest’ R package is available at: https://github.com/biodev/BioDataManifest under the GPL-3.0 license with 10.5281/zenodo.18038539. This repository also contains an R Notebook ‘manuscript_analyses.Rmd’ which contains code to reproduce the results presented in the manuscript and an editable version of the Data Manifest collection form fields.
