# Supplementary material for: Biomedical Data Manifest: A lightweight data documentation mapping to increase transparency for AI/ML
Source: Sci Data. 2026 Feb 11;13:414. doi: 10.1038/s41597-026-06670-0 (PMC13002863; doi:10.1038/s41597-026-06670-0)
Supplement: Supplementary file 1 — Supplementary File S1 [file 41597_2026_6670_MOESM1_ESM.docx]

**Biomedical Data Manifest Template**

**General Information**

1. Dataset name (abbreviation)

*In form: Enter your Answer*

2. Dataset summary

*In form: Enter your Answer*

3. Dataset Unique ID

e.g. DOI

*In form: Enter your Answer*

4. Dataset keywords

e.g. MeSH, one per line

*In form: Enter your Answer*

5. Dataset version

*In form: Enter your Answer*

6. Release Date

*In form: Please input date (M/d/yyyy)*

7. Dataset last updated

*In form: Please input date (M/d/yyyy)*

8. Link(s) to data

e.g. Links to raw and processed datasets, one per line in Markdown form: [Name](URL)
*In form: Enter your Answer*

9. Link(s) to Dataset Documentation

one per line in Markdown form: [Name](URL)

*In form: Enter your Answer*

10. Author(s) of Biomedical Data Manifest
One per line

*In form: Enter your Answer*

11. Dataset publisher(s)

e.g. The institution, organization or government responsible for publishing the dataset.  One per line
*In form: Enter your Answer*

12. Was dataset audited?  If so provide a link to dataset audit results

*In the form: yes|no,link*

13. Dataset authors

e.g. Names of the corresponding authors.  One line per name in the form: first name, last name (email; ORCHID ID (or N/A))

*In form: Enter your Answer*

14. Dataset owner

e.g. Organization that controls data / IP

*In form: Enter your Answer*

15. Summary of funding

e.g. Provide funding institution/company and list funding mechanisms.  One answer per line.
*In form: Enter your Answer*

16. Content warning

*In form: Yes, No*

17. Extension mechanisms

e.g. How to contribute to dataset

*In form: Enter your Answer*

**Uses of Data**

18. How was the dataset originally envisioned to be used?
e.g. Bridge2AI which was designed to be used for downstream AI/ML applications

*In form: Enter your Answer*

19. List of publications DOIs/URLs where dataset has been utilized including benchmarks

One per line

*In form: Enter your Answer*

20. Known concerns and limitations for future applications

e.g. is there a disease-specific use restriction as part of the consent?  One per line
*In form: Enter your Answer*

21. List of known confounding factors

e.g. Those factors that limit generalizability or otherwise act as confounders such as batch effects or composition bias.  One per line

*In form: Enter your Answer*

22. Require citation? (to use the data)

(Branching: If No, skip to Dataset Composition)

*In form: Yes, No*

23. Link to current publication(s) to cite

One per line

*In form: Enter your Answer*

**Dataset Composition**

24. What is the experimental unit referred to in this dataset?
e.g. The experimental unit is defined as the biological entity subjected
to an intervention independently of all other units, such that it is
possible to assign any two experimental units to different treatment
groups.

*In form: Enter your Answer*

25. Are there relationships between the experimental units?

e.g. Experimental units are samples that represent longitudinal timepoints from same source

*In form: Enter your Answer*

26. What is the overall sample size?

e.g. Number of experimental units

*In form: Enter your Answer*

27. Sampling Method(s)

*In form: Checkboxes below*

Convenience

Purposive

Quota

Snowball

Simple Random

Systematic Random

Stratified

Cluster

Complex/Multi-Stage

Other (Describe in free text)

28. Available datatypes/modalities for experimental units in this dataset

One per line

*In form: Enter your Answer*

29. Is there missing information in the dataset? If yes, how is it handled or represented?

*In form: Enter your Answer*

30. Data anomalies/errors

*In form: Enter your Answer*

**Ethical, Legal and Social Issues (ELSI)**

31. Is there a data license or data use agreement needed for this data?

(Branching: If No, skip to question 35)

*In form: Yes, No*

32. List any applicable license(s) governing use of the dataset

One per line

*In form: Enter your Answer*

33. List any applicable third-party intellectual property considerations

One per line

*In form: Enter your Answer*

34. List any export controls or other regulations impacting dataset access/download or storage

One per line

*In form: Enter your Answer*

35. Availability and legal considerations for external resources utilized

e.g. Indicate if some data is not available or has legal repercussions for use due to source of the data

*In form: Enter your Answer*

36. Confidentiality of data?

e.g. data that is protected by legal privilege or by doctor-patient confidentiality, data that includes the content of individuals non-public communications

*In form: Enter your Answer*

37. Inclusion criteria

*In form: Enter your Answer*

38. Is this human subjects research?

(Branching: If No, skip to Provenance and Lineage in Datasets)

*In form: Yes, No*

39. What sensitive human attributes are present?

e.g. race, gender, ethnicity or other PHI.  One per line

*In form: Enter your Answer*

40. Intentionality of sensitive human attribute collection

e.g. was some PHI collected indirectly or inferred for example using genomics to infer race or gender

*In form: Enter your Answer*

41. Known ethical, legal and social implications introduced by preprocessing

e.g. Inadvertently filtered out mostly non-white participants

*In form: Enter your Answer*

42. Known problematic proxies

e.g. variables that strongly correlate with sensitive attributes.  One per line

*In form: Enter your Answer*

43. What countries were data collected from?

One per line

*In form: Enter your Answer*

44. Was a data protection impact analysis performed?

e.g. <https://gdpr.eu/data-protection-impact-assessment-template/>

*In form: Yes, No*

45. REB/IRB approval for data collection?

(Branching: If No, skip to Provenance and Lineage in Datasets)

*In form: Yes, No*

46. Were subjects consented?

(Branching: If No, skip to Provenance and Lineage in Datasets)

*In form: Yes, No*

47. Was consent revocable?

*In form: Yes, No*

48. Considerations needed for future data updates

e.g. Can current patients opt-out or revoke consent for data use?  One per line

*In form: Enter your Answer*

**Provenance and Lineage in datasets**

49. Summarize any approaches used to validate all or part of the dataset

e.g. Targeted resequencing was performed to validate the discovery of mutations in whole exome sequencing.  One per line
*In form: Enter your Answer*

50. Describe if and how computational or manual curation was performed to generate features/variables in the dataset

e.g. Diagnostic classification where elements were extracted from EHR records and subsequently calculated/curated

*In form: Enter your Answer*

51. Please provide either a link to the Manual of Procedures (MOP) for the study or a description of the data collection methodology including Data Sources and Collection Techniques

e.g. Data Sources: Explanation of where the data originated from (surveys, sensors, web scraping, existing databases); Collection Techniques: Details on the methods used to acquire the data, such as interviews, observations, or automated processes

*In form: Enter your Answer*

52. List any specific data collection devices used and provide any relevant contextual attributes

e.g. Device specifications such as device type, IMEI/SN, software version, sensor specifications etc.  One per line

*In form: Enter your Answer*

53. Preprocessing steps/workflow
e.g. Link to code and/or manuscript.  One entry per line

*In form: Enter your Answer*

54. Data collection timeframe

*In form: Enter your Answer*

**Labeling Provenance and Lineage**

55. Were labels attributed to raw data such as images or text by expert curation, semi-automated annotation etc for use in predictive models?

(Branching: If No, skip to Maintenance and Distribution)

*In form: Yes, No*

56. Class frequency of labels

e.g. Sample frequency of a label such as responder/non-responder that was curated to be the main study outcome.  Encode in Markdown format

*In form: Enter your Answer*

57. What are the labels?

e.g. Identify the corresponding variables in the dataset.  One per line.

*In form: Enter your Answer*

58. Provide the original predictive task associated with the labels

*In form: Enter your Answer*

59. List the targets or labels with corresponding gold-standard benchmarks or proxies used for evaluation and provide details

One per line

*In form: Enter your Answer*

60. Provide the corresponding guidelines for determining target or label values.

*In form: Enter your Answer*

61. If targets or labels were rated by multiple individuals, summarize the rater agreement/disagreement

*In form: Enter your Answer*

62. Are the actual targets or labels provided in the dataset or only summarized?

*In form: Enter your Answer*

63. If software was used as part of generating targets or labels please provide details and a link

*In form: Enter your Answer*

**Maintenance and Distribution**

64. Is the dataset maintained?

e.g, process of updating, cleaning, and managing the data and its metadata over time to remain usable and reliable

(Branching: If No, end survey)

*In form: Yes, No*

65. When was the data last updated?

*In form: Please input date (M/d/yyyy)*

66. Static or dynamic versioning

*In form: Enter your Answer*

67. Number of dataset versions or releases currently available

(Branching: If 1, skip to question 70)

*In form: 1, 2, 3, 4, 5, 6, 7, 8, 9, 10+*

68. Description of the multiple versions of the dataset (If multiple exist)

Use Markdown to format.

*In form: Enter your Answer*

69. What has changed on version update?

Use Markdown to format.

*In form: Enter your Answer*

70. More versions expected?

*In form: Yes, No*

71. Who maintains the data?

*In form: Enter your Answer*

72. Contact Info

Use Markdown to format.

*In form: Enter your Answer*

73. Future update types

e.g. Genomic data will stay the same but clinical information will be updated periodically.  Use Markdown to format.

*In form: Enter your Answer*
