# Supplementary material for: Biomedical Data Manifest: A lightweight data documentation mapping to increase transparency for AI/ML
Source: Sci Data. 2026 Feb 11;13:414. doi: 10.1038/s41597-026-06670-0 (PMC13002863; doi:10.1038/s41597-026-06670-0)
Supplement: Supplementary file 2 — Supplementary Figures [file 41597_2026_6670_MOESM2_ESM.docx]

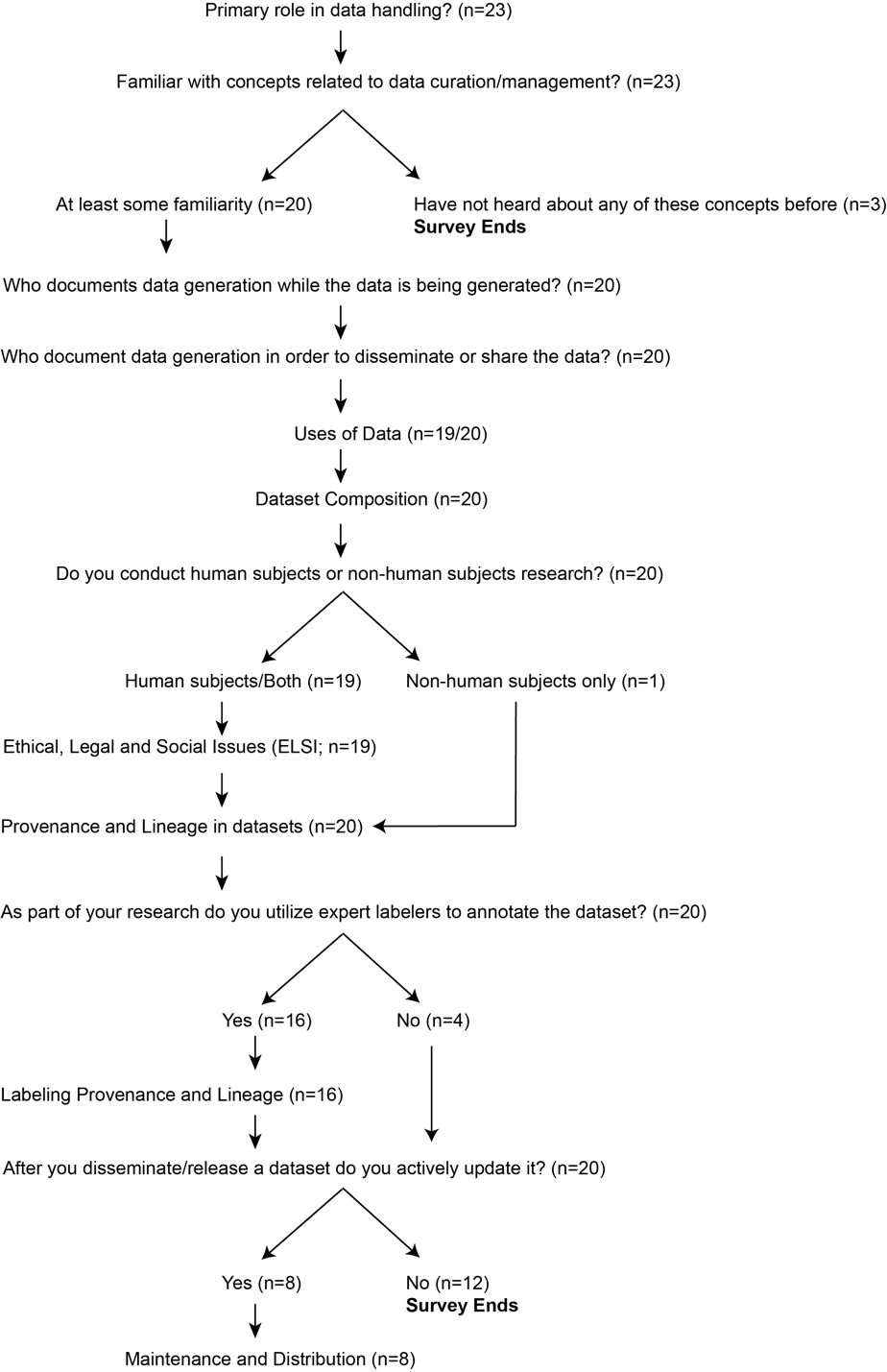


**Figure S1. Overview of the Biomedical Data Manifest survey.** Depicted is the order, flow and branching logic of both the questions and main categories of the fields/concepts that were ranked as part of the survey. The sample size in terms of the number of participants is shown in parentheses.


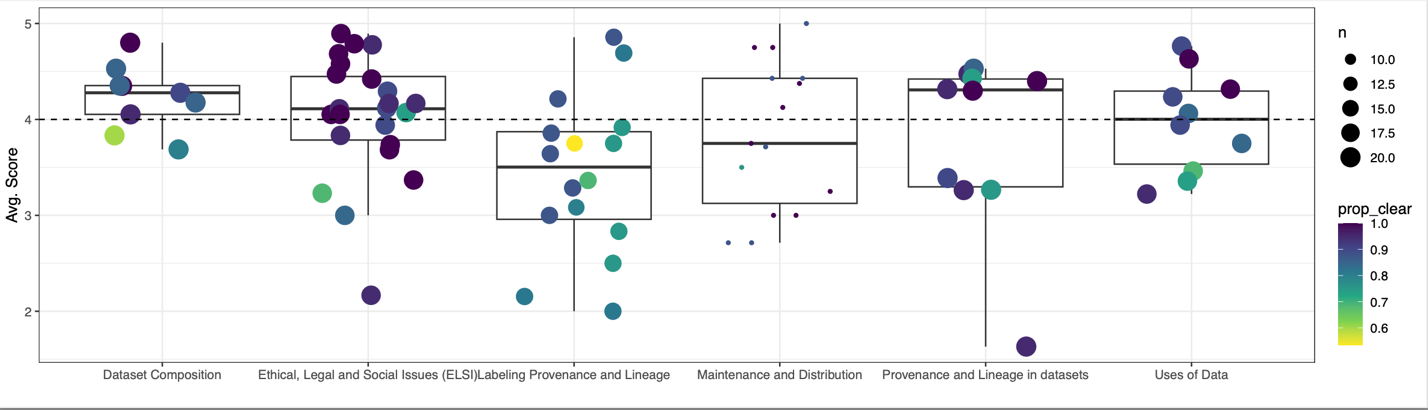


**Figure S2. Average survey scores for each of the six surveyed categories.** For each category (X-axis), the average score (Y-axis) is shown for the corresponding fields/concepts (dots) across all available survey participants. Size of the dots indicates the number of participants, while color of the dots indicates the proportion of the participants who chose a ranking as opposed to indicating that the field/concept was unclear. The dashed line indicates our cutoff of 4.0 for field/concept relevance.
